# Incidence and predictors of Woven EndoBridge (WEB) shape modification following treatment of intracranial aneurysms in a large multicenter study

**DOI:** 10.1007/s10143-025-03344-0

**Published:** 2025-02-25

**Authors:** Nimer Adeeb, Hamza Adel Salim, Basel Musmar, Assala Aslan, Christian Swaid, Miguel Cuellar, Mahmoud Dibas, Nicole M. Cancelliere, Jose Danilo Bengzon Diestro, Oktay Algin, Sherief Ghozy, Sovann V. Lay, Adrien Guenego, Leonardo Renieri, Joseph Carnevale, Guillaume Saliou, Panagiotis Mastorakos, Kareem El Naamani, Eimad Shotar, Markus Möhlenbruch, Michael Kral, Charlotte Chung, Mohamed M. Salem, Ivan Lylyk, Paul M. Foreman, Hamza Shaikh, Vedran Župančić, Muhammad U. Hafeez, Joshua Catapano, Muhammad Waqas, Muhammet Arslan, Onur Ergun, James D. Rabinov, Yifan Ren, Clemens M. Schirmer, Mariangela Piano, Anna L. Kühn, Caterina Michelozzi, Robert M. Starke, Ameer Hassan, Mark Ogilvie, Anh Nguyen, Jesse Jones, Waleed Brinjikji, Marie T. Nawka, Marios Psychogios, Christian Ulfert, Bryan Pukenas, Jan-Karl Burkhardt, Thien Huynh, Juan Carlos Martinez-Gutierrez, Muhammed Amir Essibayi, Sunil A. Sheth, Diana Slawski, Rabih Tawk, Benjamin Pulli, Boris Lubicz, Pietro Panni, Ajit S. Puri, Guglielmo Pero, Eytan Raz, Christoph J. Griessenauer, Hamed Asadi, Adnan Siddiqui, Elad I. Levy, Neil Haranhalli, David Altschul, Andrew F. Ducruet, Felipe C. Albuquerque, Robert W. Regenhardt, Christopher J. Stapleton, Peter Kan, Vladimir Kalousek, Pedro Lylyk, Srikanth Boddu, Jared Knopman, Stavropoula I. Tjoumakaris, Pascal M. Jabbour, Frédéric Clarençon, Nicola Limbucci, Vivek Yedavalli, Max Wintermark, Vitor Mendes Pereira, Aman B. Patel, Hugo H. Cuellar-Saenz, Adam A. Dmytriw

**Affiliations:** 1 Department of Neurosurgery and Interventional Neuroradiology, Louisiana State University, Shreveport, LA USA; 2 Department of Radiology, Division of Neuroradiology, Johns Hopkins Medical Center, Baltimore, MD USA; 3 Neurovascular Centre, Divisions of Therapeutic Neuroradiology and Neurosurgery, St. Michael’s Hospital, University of Toronto, Toronto, ON Canada; 4 Department of Radiology, Medical Facultyof , Ankara University, Ankara, Turkey; 5 Departments of Radiology and Neurosurgery, Mayo Clinic, Rochester, MN USA; 6 Department of Neuroradiology, Centre Hospitalier de Toulouse, Toulouse, France; 7 Department of Neuroradiology, Hôpital Universitaire Erasme, Brussels, Belgium; 8 Department of Neuroradiology, Ospedale Careggi Di Firenze, Florence, Italy; 9 Department of Neurosurgery and Neuroradiology, Weill Cornell School of Medicine, New York Presbyterian Hospitaland , New York, NY USA; 10 Department of Neuroradiology, Centre Hospitalier Vaudois de Lausanne, Lausanne, Switzerland; 11 Department of Neurosurgery, Thomas Jefferson University Hospital, Philadelphia, PA USA; 12 Department of Neuroradiology, Hôpital Pitié-Salpêtrière, Paris, France; 13 Department of Neuroradiology, Universitätsklinikum Heidelberg, Heidelberg, Germany; 14 Department of Neurosurgery, Christian Doppler University Hospital & Institute of Neurointervention, Salzburg, Austria; 15 Departments of Radiology & Neurosurgery, NYU Langone Health Center, New York, NY USA; 16 Department of Neurosurgery, University of Pennsylvania Medical Center, Philadelphia, PA USA; 17 Department of Neuroradiology, Clínica La Sagrada Familia, Buenos Aires, Argentina; 18 Department of Neurosurgery, Orlando Health Neuroscience and Rehabilitation Institute, Orlando, FL USA; 19 Department of Neuroradiology, Clinical Hospital Center ‘Sisters of Mercy’, Zagreb, Croatia; 20 Department of Neurosurgery, UTMB and Baylor School of Medicine, Houston, TX USA; 21 Department of Neurosurgery, Barrow , Neurological Institute, Phoenix, AZ USA; 22 Department of Neurosurgery, State University of New York at Buffalo, Buffalo, NY USA; 23 Deparment of Radiology, Pamukkale University, Denizli, Turkey; 24 Neuroendovascular Program, Massachusetts General Hospital, Harvard University, Boston, MA USA; 25 Department of Neuroradiology, Austin Health, Heidelberg, VIC Australia; 26 Department of Neurosurgery and Radiology, Geisinger Hospital, Danville, PA USA; 27 Department of Neuroradiology, Ospedale Niguarda Cà Granda, Milan, Italy; 28 Department of Neuroradiology, UMass Memorial Hospital, Worcester, MA USA; 29 Department of Neuroradiology, Ospedale San Raffaele, Milan, Italy; 30 Department of Neurosurgery, University of Miami, Miami, FL USA; 31 Department of Neuroradiology, Valley Baptist Neuroscience Institute, Harlingen, TX USA; 32 Deparments of Neurosurgery and Radiology, University of Alabama at Birmingham, Birmingham, AL USA; 33 Department of Neuroradiology, University Hospital of Basel, Basel, Switzerland; 34 Department of Diagnostic and Interventional Neuroradiology, University Medical Center Hamburg-Eppendorf, Hamburg, Germany; 35 Departments of Radiology and Neurosurgery, Mayo Clinic, Jacksonville, FL USA; 36 Department of Neuroradiology, University of Texas Health Science Center at Houston, Houston, TX USA; 37 Department of Neurosurgery, Montefiore Medical Center, Albert Einstein College of Medicine, Bronx, NY USA; 38 Department of Radiology, Division of Neuroimaging and Neurointervention, Stanford University School of Medicine, Stanford, CA USA; 39 Department of Neuroradiology, MD Anderson Medical Center, Houston, TX 77030 USA; 40 Department of Neurosurgery and Neurointerventional Surgery, Louisiana State University, Shreveport, LA USA

**Keywords:** Intracranial aneurysm, Embolization, Endovascular, Woven EndoBridge, WEB, Shape modification

## Abstract

The Woven EndoBridge (WEB) device is FDA-approved for the treatment of bifurcation aneurysms. Despite its wide popularity, it has been under scrutiny for its association with potential aneurysm recanalization and retreatment due to device shape modification. This study aims to analyze the shape modification rate of WEB devices and identify factors associated with this phenomenon, as well as its correlation with aneurysm retreatment. We conducted a retrospective review of the WorldWide WEB Consortium database, including adult patients treated for intracranial aneurysms with the WEB device. We assessed aneurysm occlusion using the WEB Occlusion Scale and defined WEB shape modification as a percentage reduction in the distance between two WEB markers. Logistic regression and Cox proportional hazards models were utilized to evaluate predictors of shape modification and retreatment. Kaplan–Meier curves were used to estimate the time-dependent probability of no or minor shape modification. A total of 405 patients were analyzed, with minor and major shape modification occurring in 31.4% and 10.1% of cases, respectively. Major shape modification was associated with lower rates of adequate occlusion (70.7%) compared to no or minor shape modification (86.6%) and a higher rate of retreatment (26.8% vs. 8.1%). Predictors of major shape modification included the presence of daughter sac, bifurcation aneurysms, absence of immediate flow stagnation, and a WEB width minus aneurysm width ratio ≤ 0.5. The probability of no or minor shape modification declined within the first 25 months and stabilized thereafter. WEB device shape modification is a significant predictor of aneurysm occlusion efficacy and retreatment. Recognizing the factors influencing shape modification can guide treatment decisions and follow-up protocols to improve patient outcomes.

**Supplementary Information:**

The online version contains supplementary material available at 10.1007/s10143-025-03344-0.

## Introduction

Intrasaccular flow disruption with the Woven EndoBridge (WEB) device (Microvention, Tustin, California, USA) is a promising technique for treating intracranial aneurysms. Although the FDA recently approved the device for bifurcation aneurysms, it has been in use for more than a decade. Previous studies reported a high adequate occlusion rate and a safe clinical profile [1–4].

In recent years, there has been a rising concern for the rate of aneurysm recanalization and retreatment following WEB embolization due to device shape modification [5–7]. This phenomenon corresponds to a decrease in WEB height, which can sometimes lead to aneurysmal recanalization. Although the exact cause is not well known, it is thought to be related to clot retraction during the healing process, and a high blood flow exposure could exacerbate this [5].

A better understanding of WEB shape modification and its predisposing factors can potentially lead to higher aneurysm occlusion rates. However, previous studies were limited by the small number of cases which limited the generalization of their findings and led to contradictory findings in shape modification rate and relevance to aneurysm recurrence and retreatment [5, 7–9].

The WorldWideWEB consortium was established as the most extensive global retrospective multicenter WEB registry. In the present study, we performed a sub-analysis of the consortium that investigates the shape modification rate of implanted WEB devices and the factors associated with this phenomenon. We also aimed to study the correlation between WEB shape modification and aneurysm retreatment.

## Materials and methods

### Patient population

A retrospective review of the WorldWide WEB Consortium, a synthesis of prospectively maintained databases at academic institutions in North America, South America, Europe, and Australia, was performed to identify patients with intracranial aneurysms treated with WEB device between 2011 and 2022. Selection of aneurysms for WEB treatment was based on clinical and anatomical criteria, including aneurysm size, and wide-neck morphology. Decisions were made at the discretion of the treating physician.

The following information was collected: patient demographics, aneurysm characteristics, antiplatelet regimen, procedural details, complications, angiographic and functional outcomes. Only adult patients (age > 18 years) with available aneurysm measurements, imaging follow-up, and shape modification rate were included in this study. Both ruptured and unruptured aneurysms in all locations were included. Both bifurcation and sidewall aneurysms were included. Institutional Review Board approval was obtained at all centers included in the consortium.

### Angiographic and functional outcomes

The angiographic outcome was assessed using digital subtraction angiography (DSA). Aneurysm occlusion after treatment, both immediately and at last follow-up, was categorized using the Raymond Roy Occlusion Classification (RROC): complete occlusion (class 1), residual neck (class 2), and residual aneurysm (class 3). Adequate occlusion was defined as either complete occlusion or residual neck without a residual aneurysm. Other angiographic outcomes included immediate blood flow stagnation, patency of branches arising from the aneurysm at last follow-up, and aneurysm recurrence. Immediate blood flow stagnation was defined as a significant slowing of blood flow into the aneurysm sac immediately following WEB device deployment. This phenomenon indicates effective disruption of intra-aneurysmal flow but does not necessarily correlate with complete aneurysm occlusion at follow-up.

WEB device shape modification was defined as the percentage of reduction in the distance between the two WEB markers (distal and proximal) between the initial procedure DSA and imaging at last follow-up. It was then classified into no shape modification (0%), minor shape modification (< 50%), and major shape modification (> 50%). A similar classification was also adopted in previous studies [5]. Functional outcome was assessed using the modified Rankin Scale (mRS) at last follow-up.

### Complications

Thromboembolic complications occurring from the date of the procedure up to the last follow-up were recorded. Intra-procedural thromboembolic complications were identified on DSA as either thrombus formation, slow filling of a previously normal filling vessel, or vessel occlusion. Post-procedural thromboembolic complications were identified using a combination of clinical and radiographic findings. Post-procedural imaging was performed at the discretion of the individual institutions. Routine screening for clinically silent infarcts was not consistently performed. Post-procedural imaging obtained to detect a symptomatic ischemic stroke could include any combination of a non-contrast computed tomography (CT), CT angiography, or magnetic resonance imaging. Only ischemic strokes in the territory of the treated vessel were included. An ischemic complication was considered symptomatic if there were patient-reported symptoms or clinical signs attributable to thromboembolism; this included transient or resolving signs and symptoms. Complications were considered permanent if still present at 3-month follow-up.

Hemorrhagic complications were identified intra-operatively as contrast extravasation on DSA or post-procedure imaging. Hemorrhagic complications occurring from the time of the procedure up until the last follow-up were included. Hemorrhages were counted as symptomatic if the patient-reported symptoms or demonstrated signs attributable to hemorrhage.

### Statistical analysis

Statistical analysis was performed using R software (version 4.3.1, R Foundation for Statistical Computing, Vienna, Austria). Categorical variables were presented as frequencies and percentages and compared using the Chi-square test, while continuous variables were presented as median (IQR) and compared using the Mann–Whitney U test.

The utilization of Kaplan–Meier curves was employed in order to examine the likelihood of no or minor shape modification. The log-rank tests were employed to assess and compare the survival curves across the various groups. To find out how baseline predictors affected the rate of device shape modification at the last follow-up, a univariable Cox proportional hazards ratio was used. Multivariable logistic regression was used to determine the relationship between major shape modification and outcomes of interest. All those with *p* < 0.1 were included in multivariable regression models to determine the relationship of our covariates of interest to the outcomes. Forced inclusion of some key variables was done based on scientific rationale. Results were deemed statistically significant if *p* < 0.05. Lastly, we built receiver-operator characteristic (ROC) curve and employed the Youden index to determine the optimal cutoff point for the “WEB width minus aneurysm width”, Aspect ratio, and height to width ratio to predict “Major shape modification”.

## Results

### Baseline characteristics

In this multicenter study, a total of 405 patients were evaluated for the incidence and predictors of WEB shape modification following treatment of intracranial aneurysms. Minor and major shape modification occurred in 127 (31.4%) and 41 (10.1%) of cases, respectively. Among these, females represented a majority with a total of 298 cases (73.6%). The median age at which patients presented was 61 years (IQR: 53 to 68), with those experiencing major shape modification being slightly younger at a median of 58 years (IQR: 49 to 64), a difference that was statistically significant (*p* = 0.017). The presentation of intracranial aneurysms varied, with incidental/asymptomatic cases being the most common (218 patients, 57.4%). The majority of patients presented with unruptured aneurysms (313 patients, 77.3%) (Table 1).

**Table 1:** Baseline characteristics of patients included in the study

Variables		No/minor shape modification	Major shape modification	Total (*n* = 405)	*P*-value
		(*n* = 364)	(*n* = 41)		
Sex	**Female**	265 (72.8)	33 (80.5)	298 (73.6)	0.384
	**Male**	99 (27.2)	8 (19.5)	107 (26.4)	
Age	**Median (IQR)**	61.5 (53.0 to 68.0)	58.0 (49.0 to 64.0)	61.0 (53.0 to 68.0)	**0.017**
Smoking		104 (28.8)	17 (41.5)	121 (30.1)	0.135
Presentation	**CN Palsy**	6 (1.8)	0 (0.0)	6 (1.6)	0.94
	**Headache/Dizziness**	49 (14.5)	6 (14.6)	55 (14.5)	
	**Incidental/Asymptomatic**	192 (56.6)	26 (63.4)	218 (57.4)	
	**Recurrence**	1 (0.3)	0 (0.0)	1 (0.3)	
	**Ruptured aneurysm**	84 (24.8)	8 (19.5)	92 (24.2)	
	**Seizures**	1 (0.3)	0 (0.0)	1 (0.3)	
	**Weakness/Numbness**	6 (1.8)	1 (2.4)	7 (1.8)	
Ruptured aneurysm	**No**	280 (76.9)	33 (80.5)	313 (77.3)	0.511
	**Yes**	84 (23.1)	8 (19.5)	92 (22.7)	
Hunt Hess Grade	**Median (IQR)**	3.0 (1.0 to 3.0)	1.5 (1.0 to 3.0)	3.0 (1.0 to 3.0)	0.135
Pretreatment mRS	**3–5**	19 (5.5)	2 (4.9)	21 (5.4)	1
	**0–2**	328 (94.5)	39 (95.1)	367 (94.6)	
Location	**Anterior cerebral artery**	117 (32.1)	7 (17.1)	124 (30.6)	**0.003**
	**Internal carotid artery**	52 (14.3)	2 (4.9)	54 (13.3)	
	**Vertebrobasilar artery**	69 (19.0)	6 (14.6)	75 (18.5)	
	**Middle cerebral artery**	126 (34.6)	26 (63.4)	152 (37.5)	
Bifurcation		309 (84.9)	35 (85.4)	344 (84.9)	1
Multiple aneurysms		118 (32.4)	16 (39.0)	134 (33.1)	0.498
Prior treatment		18 (5.5)	2 (5.7)	20 (5.5)	1
Neck (mm)	**Median (IQR)**	4.0 (3.3 to 5.5)	4.5 (3.7 to 6.0)	4.1 (3.3 to 5.5)	**0.046**
Height (mm)	**Median (IQR)**	6.0 (5.0 to 8.0)	6.0 (5.0 to 7.7)	6.0 (5.0 to 7.9)	0.97
Width (mm)	**Median (IQR)**	5.6 (4.4 to 7.2)	6.8 (5.0 to 8.0)	5.7 (4.5 to 7.4)	**0.023**
Aspect	**Median (IQR)**	1.5 (1.1 to 1.9)	1.3 (1.2 to 1.4)	1.4 (1.1 to 1.9)	**0.028**
Maximum diameter (mm)	**Median (IQR)**	7.0 (6.0 to 8.0)	7.0 (6.0 to 9.0)	7.0 (6.0 to 9.0)	0.303
Width to Neck ratio	**Median (IQR)**	1.3 (1.1 to 1.6)	1.3 (1.1 to 1.6)	1.3 (1.1 to 1.6)	0.989
Height to width ratio	**Median (IQR)**	1.1 (0.9 to 1.3)	1.0 (0.8 to 1.1)	1.1 (0.9 to 1.3)	**0.004**
WEB width minus aneurysm width	**Median (IQR)**	0.9 (0.2 to 1.5)	0.5 (0.0 to 1.0)	0.9 (0.1 to 1.4)	**0.08**
Height to width ratio	** < 1.2**	218 (59.9)	38 (92.7)	256 (63.2)	** < 0.001**
	** > 1.2**	146 (40.1)	3 (7.3)	149 (36.8)	
WEB width minus aneurysm width	** > 0.5**	164 (45.1)	7 (17.1)	171 (42.2)	** < 0.001**
	** ≤ 0.5**	200 (54.9)	34 (82.9)	234 (57.8)	
Aspect ratio	** < 1.5**	185 (54.3)	31 (79.5)	216 (56.8)	**0.004**
	** > 1.5**	156 (45.7)	8 (20.5)	164 (43.2)	
Daughter sac		94 (27.9)	15 (39.5)	109 (29.1)	0.193
Branch from aneurysm		49 (13.6)	4 (9.8)	53 (13.2)	0.659

Most patients had a pre-treatment modified Rankin Scale (mRS) score of 0–2, comprising 328 (94.5%) and 39 (95.1%) in the minor or no shape modification and major shape modification groups, respectively.

Most aneurysms were bifurcation aneurysms (84.9%) and were more frequently located in the middle cerebral artery (MCA) (37.5%), anterior cerebral artery (30.6%), and the vertebrobasilar artery (18.5%). The median maximum aneurysm diameter, height, width, and neck size were 7 mm, 6 mm, 5.7 mm, and 4.1 mm, respectively. A daughter sac was present in 29.1% of aneurysms while an incorporated arterial branch was present in 13.2% of aneurysms. A prior treatment was done in 5.5% of aneurysms (Table 1).

The median height to width ratio was significantly different between groups, with the minor or no shape modification group having a higher ratio (1.1 (IQR: 0.9 to 1.3)) compared to the major shape modification group (1 (IQR: 0.8 to 1.1)) (*p* = 0.004). The WEB width minus aneurysm width showed a median difference of 0.9 mm (IQR: 0.1 to 1.4), with a less pronounced difference in the major shape modification group (*p* = 0.08). In addition, the median Aspect ratio showed a significant difference between the two groups, with the minor or no shape modification group having a higher ratio (1.5 (IQR: 1.1 to 1.9) compared to the major shape modification group (1.3 (IQR: 1.2 to 1.4)) (*p* = 0.028).

### Treatment outcomes

Most procedures were performed through femoral access (83.5%). The median follow-up imaging was longer in the major shape modification group with a median of 19.5 months (IQR: 8 to 26.7 months) compared to 10.0 months (IQR: 6.0 to 16.0 months) in the minor or no shape modification group (*p* = 0.001). Immediate flow stagnation was more prevalent in the no or minor shape modification group at 90.7% versus 70.7% in the major shape modification group (*p* < 0.001) (Table 2).

**Table 2:** Treatment outcomes of patients included in the study

Variables		No or minor shape modification	Major Shape modification	Total	*P*-value
Access	**Femoral**	299 (82.1)	39 (95.1)	338 (83.5)	0.058
	**Radial**	65 (17.9)	2 (4.9)	67 (16.5)	
Thromboembolic complications (TE)		23 (6.3)	2 (4.9)	25 (6.2)	0.983
TE timing	**Intraoperative**	15 (68.2)	0 (0.0)	15 (62.5)	0.253
	**Postoperative**	7 (31.8)	2 (100.0)	9 (37.5)	
TE Duration	**Permanent**	5 (21.7)	0 (0.0)	5 (20.0)	1
	**Transient**	18 (78.3)	2 (100.0)	20 (80.0)	
Hemorrhagic complications		8 (2.2)	1 (2.5)	9 (2.2)	1
Hemorrhagic complications timing	**Intraoperative**	4 (50.0)	1 (100.0)	5 (55.6)	1
	**Postoperative**	4 (50.0)	0 (0.0)	4 (44.4)	
Hemorrhagic complications duration	**Permanent**	2 (25.0)	0 (0.0)	2 (22.2)	1
	**Transient**	6 (75.0)	1 (100.0)	7 (77.8)	
Other complications		15 (5.7)	2 (6.9)	17 (5.8)	1
Other complications type	**Air Embolization**	1 (7.1)	0 (0.0)	1 (6.2)	0.347
	**Deployment issues**	8 (57.1)	1 (50.0)	9 (56.2)	
	**Puncture site hematoma/pseudoaneurysm**	4 (28.6)	0 (0.0)	4 (25.0)	
	**Vascular Dissection**	1 (7.1)	1 (50.0)	2 (12.5)	
last clinical follow-up	**Median (IQR)**	12.0 (6.0 to 18.0)	18.0 (8.9 to 36.5)	12.3 (6.0 to 20.4)	**0.006**
Last mRS score	**0**	270 (78.7)	37 (90.2)	307 (79.9)	0.497
	**1**	36 (10.5)	3 (7.3)	39 (10.2)	
	**2**	14 (4.1)	0 (0.0)	14 (3.6)	
	**3**	13 (3.8)	0 (0.0)	13 (3.4)	
	**4**	5 (1.5)	0 (0.0)	5 (1.3)	
	**5**	1 (0.3)	0 (0.0)	1 (0.3)	
	**6**	4 (1.2)	1 (2.4)	5 (1.3)	
Last imaging follow-up	**Median (IQR)**	10.0 (6.0 to 16.0)	19.5 (8.0 to 26.7)	11.0 (6.0 to 17.0)	**0.001**
Immediate flow stagnation		330 (90.7)	29 (70.7)	359 (88.6)	** < 0.001**
Immediate occlusion	**1**	72 (19.8)	2 (4.9)	74 (18.3)	**0.02**
	**2**	76 (20.9)	6 (14.6)	82 (20.2)	
	**3**	216 (59.3)	33 (80.5)	249 (61.5)	
Immedaite Raymond Roy	**1**	90 (24.7)	4 (9.8)	94 (23.2)	0.052
	**2**	74 (20.3)	7 (17.1)	81 (20.0)	
	**3**	200 (54.9)	30 (73.2)	230 (56.8)	
Last follow-up occlusion	**1**	219 (62.4)	12 (29.3)	231 (58.9)	**< 0.001**
	**2**	82 (23.4)	16 (39.0)	98 (25.0)	
	**3**	50 (14.2)	13 (31.7)	63 (16.1)	
Last follow-up RR	**1**	211 (60.1)	12 (29.3)	223 (56.9)	**< 0.001**
	**2**	93 (26.5)	17 (41.5)	110 (28.1)	
	**3**	47 (13.4)	12 (29.3)	59 (15.1)	
Adequate of occlusion at last follow-up	**Adequate**	304 (86.6)	29 (70.7)	333 (84.9)	**0.014**
	**Inadequate**	47 (13.4)	12 (29.3)	59 (15.1)	
Retreatment		29 (8.1)	11 (26.8)	40 (10.0)	** < 0.001**
Retreatment type	**Clipping**	3 (10.3)	1 (9.1)	4 (10.0)	1
	**Endovascular techniques**	26 (89.7)	10 (90.9)	36 (90.0)	

**Fig. 1:**
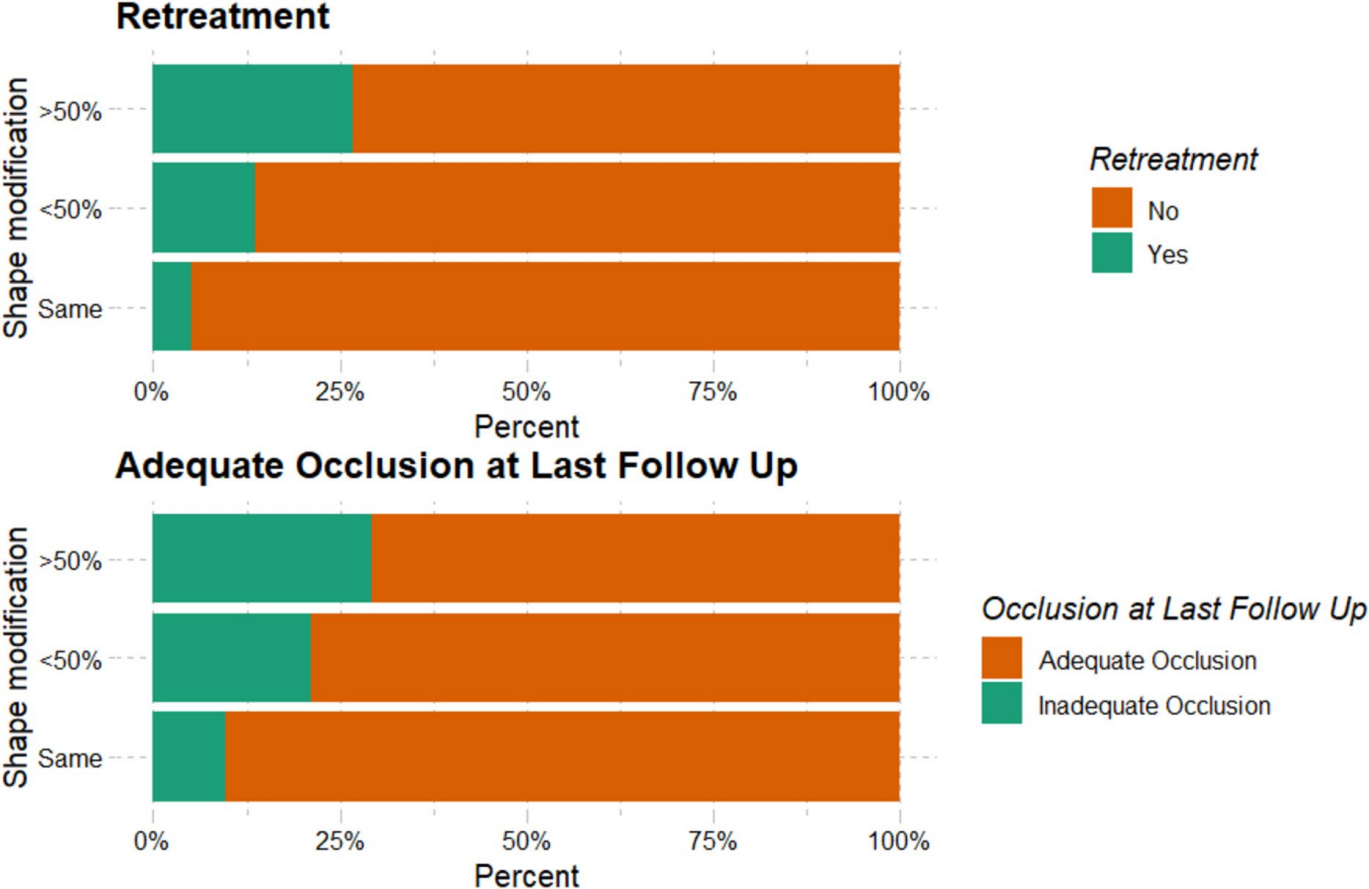
Bar chart comparing retreatment, and adequate occlusion between major shape modification, minor shape modification, or no shape modification

There were significant differences in retreatment rates, with 11/40 (26.8%) patients in the major shape modification group undergoing retreatment compared to 29/359 (8.1%) in the minor or no shape modification group (*p* < 0.001) (Fig. 1).

**Fig. 2:**
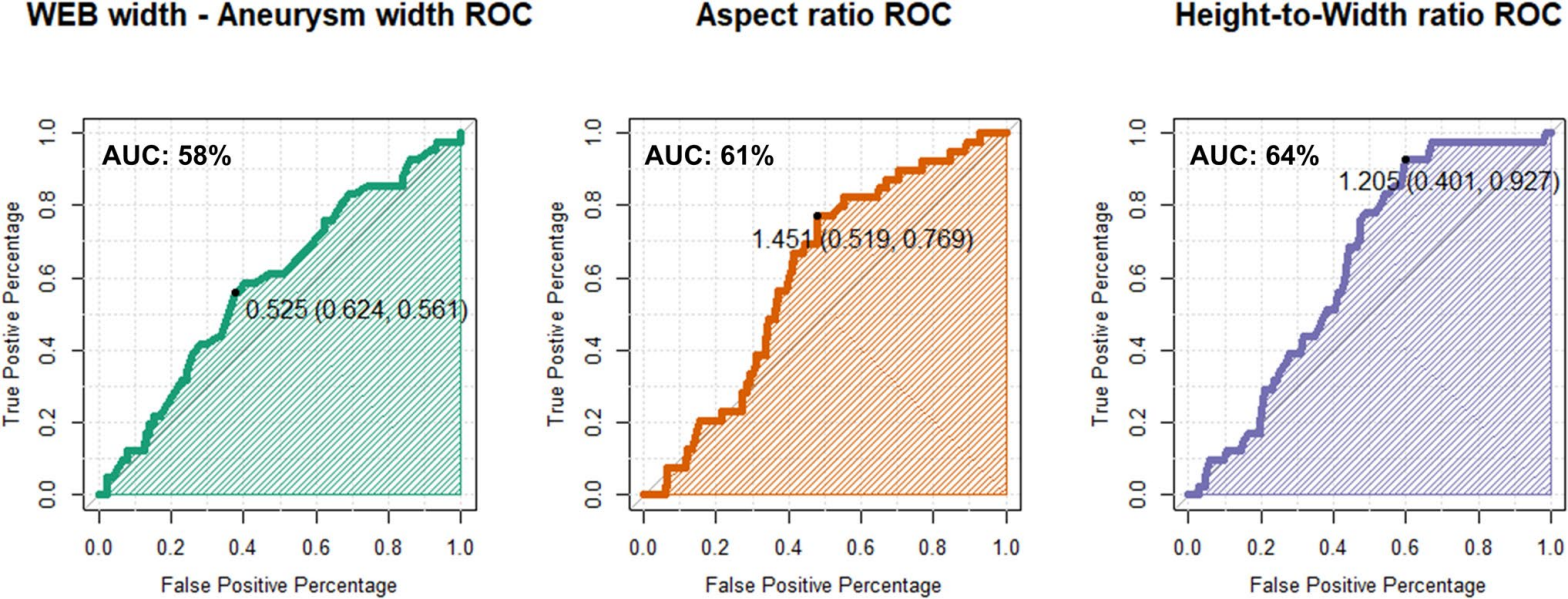
ROC curves for WEB width minus aneurysm width, aspect ratio, height to width ratio

At the final imaging follow-up, adequate occlusion was achieved less frequently in the major shape modification group (70.7%) compared to the minor or no shape modification group (86.6%), with the difference being statistically significant (*p* = 0.014) (Fig. 1). No significant difference was found between the two groups in terms of hemorrhagic complications (*p* = 1) or thromboembolic complications (*p* = 0.983). The cut-off points for “WEB width minus aneurysm width”, Aspect ratio, and height to width ratio to predict “Major shape modification” were determined using the Youden index, as documented in the ROC curves in Fig. 2.

**Fig. 3:**
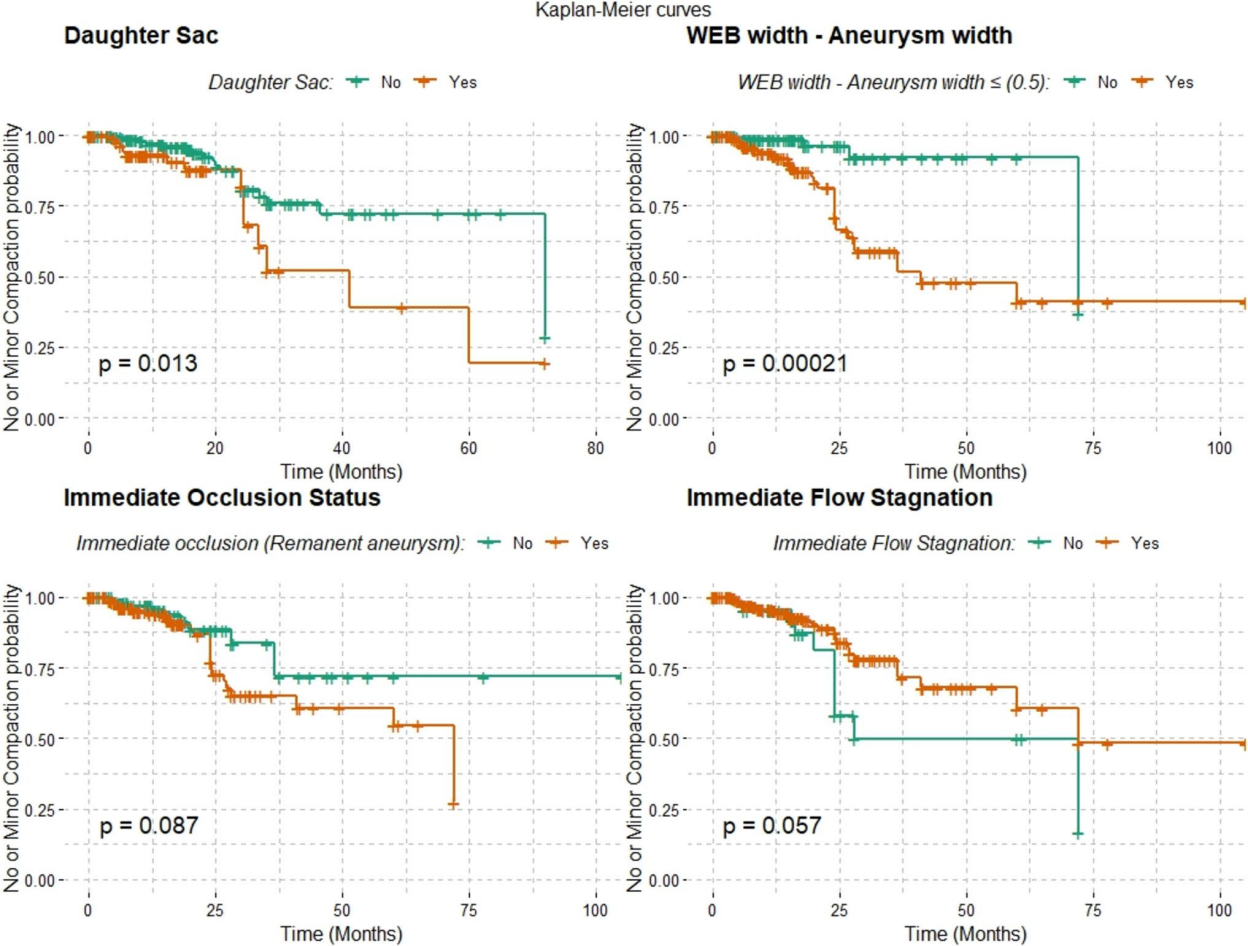
Kaplan–Meier survival Curves comparing the probability of no or minor shape modification occurrence over time across various conditions

The Kaplan–Meier survival analyses were conducted to compare the probability of no or minor shape modification occurrence over time across various conditions. The presence of a daughter sac was found to significantly influence the likelihood of no or minor shape modification (*p* = 0.013). Moreover, when considering the relationship between the WEB width minus aneurysm width ≤ 0.5 and shape modification, the analyses revealed a highly significant association, with a *p*-value of 0.00021. However, the attainment of immediate occlusion status post-treatment did not demonstrate a statistically significant correlation with the incidence of no or minor shape modification (*p* = 0.087). Similarly, patients exhibiting immediate flow stagnation after treatment showed no significant correlation with the incidence of no or minor shape modification (Fig. 3).

**Fig. 4:**
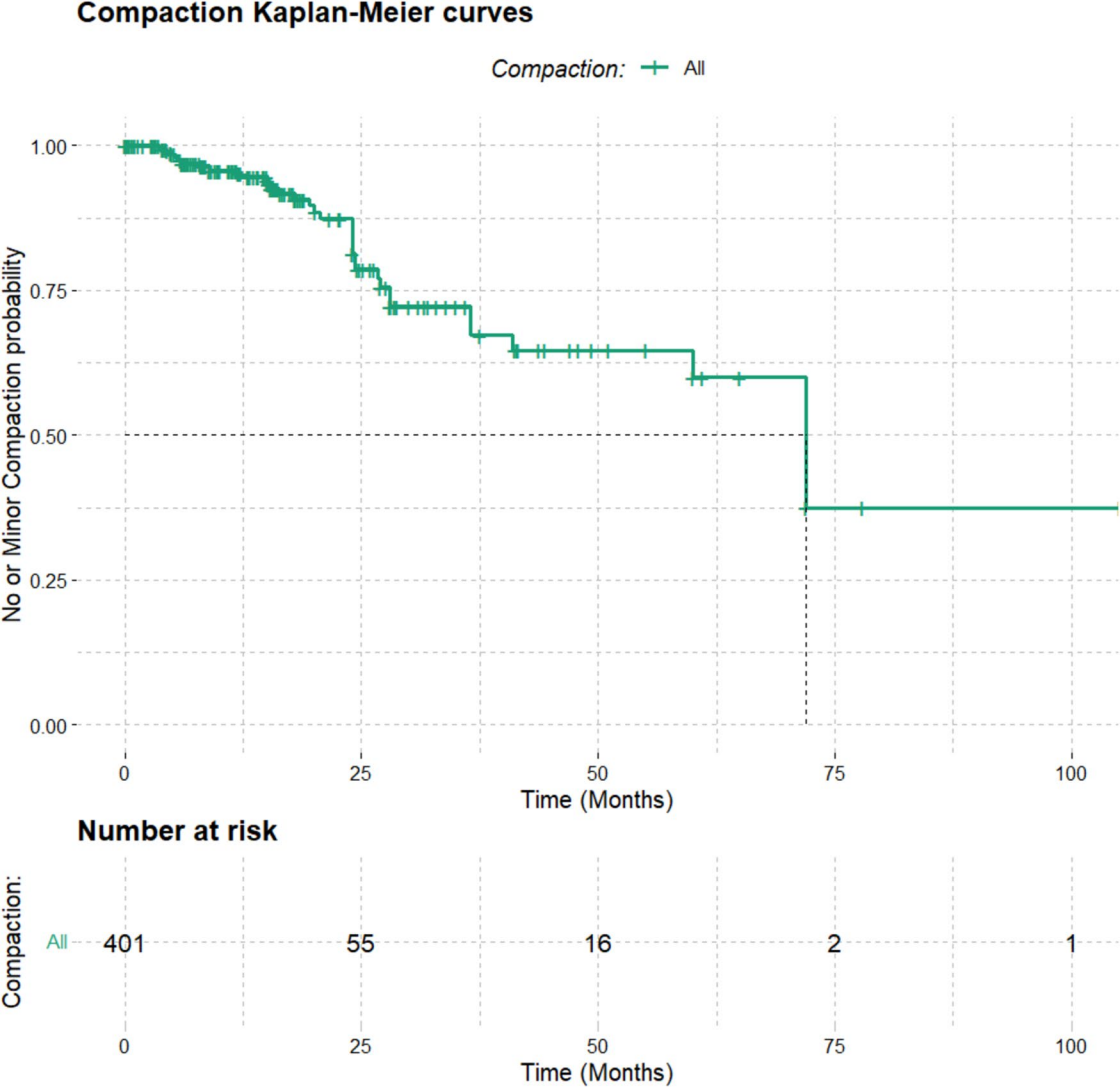
Overall Kaplan–Meier curve for the entire cohort demonstrating the time-dependent probability of no or minor shape modification following aneurysm treatment

Lastly, the overall Kaplan–Meier curve for the entire cohort demonstrates the time-dependent probability of no or minor shape modification following aneurysm treatment. Initially, all 405 patients were at risk, with a 100% probability of no or minor shape modification. However, within the first 25 months, a notable decline in this probability indicates that shape modification events were most frequent during this early period. As time progressed beyond 25 months, the decline in the probability of no or minor shape modification tapered off, implying a reduced rate of these events. This trend continued up to 100 months, where the data showed the probability stabilizing as the number of patients at risk diminished, concluding with only one patient at risk by this final time point (Fig. 4).

### Multivariable logistic regression

After adjusting the model to sex, age, smoking status, pretreatment mRS, location, aneurysm dimensions, immediate inadequate occlusion, and rupture aneurysm status, major shape modification was found to be a significant predictor of retreatment (OR: 4.93; CI: 1.74 to 13.8, *p* < 0.001) (Table 3).

**Table 3:** Adjusted multivariable logistic regression analysis of major shape modifications and their association with outcomes and post-procedural complications

Outcome	OR	95% CI	*P*-value
Retreatment	4.9	1.74, 13.8	< 0.001
Thromboembolic	0.6	0.08, 2.28	0.47
Intracranial hemorrhage	1	0.04, 8.53	0.99
Inadequate Occlusion at last follow up*	2.3	0.92, 5.44	0.07

^*^versus complete or remanent neck

The analysis was adjusted for Sex, age, smoking status, pretreatment mRS, location, aneurysm dimensions, immediate inadequate occlusion, rupture aneurysm status

### Multivariable cox hazards proportional regression model

In the multivariable Cox regression model, several predictors were found to be significantly associated with the occurrence of major shape modification in patients. These predictors included daughter sac (HR: 2.75; CI 1.20 to 6.29, *p* = 0.016), bifurcation aneurysms (HR: 0.18; CI: 0.04 to 0.9, *p* = 0.036), immediate flow stagnation (HR: 0.31; CI: 0.12 to 0.79, *p* = 0.014), and WEB width minus aneurysm width ratio ≤ 0.5 (HR: 4.57; CI: 1.59 to 13.2, *p* = 0.005) (Table 4).

**Table 4:** Cox Proportional hazards regression model

Characteristic	Univariable cox regression		Multivariable cox regression	
	HR (95% CI)	*p*-value	HR (95% CI)	*p*-value
Age	0.97 (0.94 to 1.0)	0.021	0.98 (0.94 to 1.01)	0.19
Sex: Male	0.98 (0.44 to 2.15)	0.95		
Smoking	2.68 (1.42 to 5.05)	0.002	1.43 (0.67 to 3.04)	0.36
Pretreatment mRS: 0–2	0.99 (0.24 to 4.14)	> 0.99		
Anterior cerebral artery	*Reference*		—	
Internal carotid artery	1.37 (0.28 to 6.73)	0.7	0.52 (0.07 to 3.98)	0.53
Vertebrobasilar artery	1.94 (0.65 to 5.78)	0.24	1.09 (0.31 to 3.86)	0.89
Middle cerebral artery	2.29 (0.99 to 5.31)	0.053	1.87 (0.75 to 4.66)	0.18
Ruptured aneurysm	1.22 (0.56 to 2.66)	0.61	1.32 (0.52 to 3.38)	0.56
Prior Treatment	0.88 (0.21 to 3.68)	0.86		
Multiple Aneurysms	1.10 (0.58 to 2.07)	0.77		
Bifurcation	0.60 (0.25 to 1.45)	0.26	0.18 (0.04 to 0.90)	0.036
Branch from aneurysm	0.32 (0.11 to 0.94)	0.039	0.72 (0.24 to 2.18)	0.56
Daughter sac	2.18 (1.13 to 4.18)	0.02	2.75 (1.20 to 6.29)	0.016
Neck	1.07 (0.88 to 1.31)	0.48		
Max diameter	1.01 (0.89 to 1.14)	0.89		
Width	1.06 (0.94 to 1.19)	0.33		
Height	0.97 (0.85 to 1.10)	0.6		
Height/width > 1.2	0.14 (0.04 to 0.46)	0.001	0.21 (0.06 to 0.81)	0.024
Aspect > 1.5	0.41 (0.19 to 0.90)	0.026	0.75 (0.30 to 1.88)	0.54
Dome/Neck	1.14 (0.56 to 2.33)	0.72		
Access				
Femoral	—			
Radial	1.06 (0.24 to 4.61)	0.94		
Immediate flow stagnation	0.51 (0.26 to 1.01)	0.053	0.31 (0.12 to 0.79)	0.014
WEB width—Aneurysm width ≤ 0.5	4.03 (1.78 to 9.11)	< 0.001	4.57 (1.59 to 13.2)	0.005
Immediate occlusion: Remanent	1.83 (0.91 to 3.66)	0.088	1.35 (0.56 to 3.27)	0.5
Aneurysm*				

^*^versus complete or remanent neck

### No shape modification and minor shape modification (< 50%)

The Cox proportional hazards regression model was used to determine if the factors associated with major shape modification also applied to minor shape modification (< 50%) when compared to no shape modification. The model showed that most variables significant in the major shape modification group did not maintain their significance in this comparison. For instance, smoking status remained a significant predictor in both univariable (HR, 1.87; 95% CI: 1.26–2.77; *p* = 0.002) and multivariable (HR, 1.83; 95% CI: 1.17–2.86; *p* = 0.008) analyses. However, other variables such as WEB width minus aneurysm width ≤ 0.5 (HR, 1.12; 95% CI: 0.72–1.73; *p* = 0.62), age (HR, 0.99; 95% CI: 0.97–1.00; *p* = 0.14), secondary aneurysm location (HR, 1.33; 95% CI: 0.83–2.12; *p* = 0.23), ruptured aneurysm status (HR, 1.18; 95% CI: 0.72–1.94; *p* = 0.51), and aneurysm neck size (HR, 0.97; 95% CI: 0.86–1.10; *p* = 0.68) were not significant in the comparison between no and minor shape modifications (Supplementary Table 1).

The treatment outcomes were analyzed for patients with no shape modification and those with minor shape modification. The analysis indicated no significant differences in thromboembolic (6.3% vs. 6.3%, *p* > 0.99) and hemorrhagic complications (2.1% vs. 2.4%, *p* > 0.99) between the groups. Retreatment was required significantly more often in the minor shape change group (14% vs. 5.1%, *p* = 0.004) (Supplementary Table 2). The adjusted multivariable logistic regression revealed that minor shape modification has a significant association with retreatment (OR, 4.04; 95% CI: 1.29–14.8; *p* = 0.022) and inadequate occlusion at last follow-up (OR, 3.95; 95% CI: 1.69–9.91; *p* = 0.002) (Supplementary Table 3).

## Discussion

In the present study, we examined the rate of WEB device shape modification in a large international retrospective cohort. Minor and major shape modification occurred in 31.4% and 10.1% of cases, respectively. Major shape modification was associated with a significantly higher rate of incomplete aneurysm occlusion at last follow-up and a higher retreatment rate compared to no or minor shape modification. Cox analysis underscored the importance of WEB width minus aneurysm width, the presence of daughter sacs, bifurcation aneurysms, and immediate flow stagnation in predicting shape modification events. Moreover, multivariable logistic regression revealed that major shape modification was found to be a significant predictor of retreatment rates.

When comparing patients with no shape modification to those with minor shape modification, the significant predictors of major shape modification largely lost their significance. Variables such as age, secondary aneurysm location, ruptured aneurysm status, and aneurysm neck size, which were significant in the major shape modification group, were not significant in this comparison. Additionally, the WEB width minus aneurysm width ratio did not maintain its significance between minor and no shape modification groups (HR 1.12, 95% CI 0.72–1.73, *p* = 0.62).

Pierot et al. reported the 1-year [10], 2-years [4] and 3-years [11] follow-up of combined data from the two WEBCAST (WEB Clinical Assessment of Intrasaccular Aneurysm Therapy) and French Observatory trials in what was considered the largest multicenter WEB database. In those studies, aneurysm retreatment rate increased from 7.2% at 1 year to 9.2% at 2 years and 11.4% at 3 years after device implantation [4, 10, 11]. In the WEB-IT (WEB Intrasaccular Therapy) trial, adequate occlusion was achieved in 85.6% of cases at 1-year follow-up. Between the 6-months and 1 year follow-up, 11.5% of aneurysms showed some degree of recanalization. Retreatment was needed in 9.8% of cases at 1 year [3].

The concern for intra-saccular WEB shape modification and the consequent increased risk of recanalization and the need for retreatment was first raised by Cognard and Januel [7], and it was further evaluated in other small-sized studies [5, 6, 8, 9]. This phenomenon is defined as a decrease in the height of the device owing to the deepening of the proximal and distal concave device recesses during follow-up [5]. Because both the proximal marker (near the aneurysm neck) and the distal marker (near the aneurysm apex) move toward the center of the device with time, one hypothesis is that the mechanism responsible for this phenomenon is likely associated with clot organization and retraction [1]. However, this issue or its precursors was not addressed in the large WEB trials leading to absence or generalizable findings [3, 4, 10].

One prospective study of 51 aneurysms treated with the WEB device showed that during a total follow-up period of 5 years, shape modification was observed in 72.9% of cases. However, shape modification did not correlate with adequate occlusion rates in that series [1]. Conversely, a study by Caroff et al. demonstrated that the absence of WEB shape modification was almost a guarantee of an adequate occlusion at follow-up in the 12 aneurysms in their cohort with no WEB shape modification [5]. In the present study, aneurysms with WEB shape modification had a significantly lower rate of adequate occlusion, as no or minor shape modification (< 50%) and major shape modification (> 50%) had adequate occlusion rates of 86.6% and 70.7%, respectively. Major shape modification also led to a significantly higher rate of aneurysm retreatment (26.8%) compared to no or minor shape modification (8.1%) at last follow-up.

Few previous studies have suggested that oversizing the WEB width by 1–2 mm might significantly lower rate of WEB shape modification, with no significant correlation with device height [5, 12]. However, those studies were limited by a small number of patients. In the present study, we determined that oversizing the WEB width by 0.5 mm or more is a significant predictor of no or minor shape modification. Conversely, choosing a WEB smaller than the recommended size appears to result in ‘compression,’ a phenomenon associated with inadequate occlusion. This specific finding highlights the need for careful device sizing but cannot be generalized to all cases of WEB shape modification, as shape modification may also result from other mechanisms such as clot retraction and high arterial inflow.

Contrary to the findings of Caroff et al., who reported that WEB shape modification mostly occurred in the early stages after device implantation and it stabilized after 9 months [5], we found that shape modification, in fact, may continue until 25 months of follow-up, stabilizing thereafter.

Our study found the presence of daughter sac to affect shape modification. While the exact mechanism remains speculative, the daughter sac’s irregular morphology could result in increased mechanical stress or differential blood flow patterns, which might accelerate or amplify the shape modification process.

### Limitations

The primary limitations of the current study include its retrospective design and variability in the management of patients across centers. Retrospective studies are subject to incomplete datasets, selection bias, and unidentified confounders. The inclusion of multiple institutions improves the generalizability of the findings but introduces variability in aneurysm measurement, patient management, follow-up protocol, and assessment of aneurysm occlusion or shape modification status, among others. Additionally, while this study identified a strong association between undersized WEB devices and ‘compression’ leading to inadequate occlusion, this observation cannot be generalized to all cases of WEB shape modification. Other factors such as clot retraction and high arterial inflow may also play significant roles in shaping outcomes and warrant further investigation. Also, major shape modification is more likely to occur with longer follow-up durations, which could influence the observed differences between groups. Furthermore, we recognize that a stricter definition of minor shape modification (e.g., 10–50%) might offer a clearer distinction, as the current definition inherently includes cases with no shape modification (0%).

## Conclusion

The current study highlights major WEB device shape modification as a significant determinant of aneurysm occlusion efficacy and retreatment necessity, emphasizing the importance of its consideration in post-embolization patient care and follow-up protocols.

## Supplementary Information

Below is the link to the electronic supplementary material.

ESM 1 (DOCX 13.4 KB)

ESM 2 (DOCX 20.6 KB)

## Data Availability

No datasets were generated or analysed during the current study.
