# Supplementary material for: Incidence and predictors of Woven EndoBridge (WEB) shape modification following treatment of intracranial aneurysms in a large multicenter study
Source: Neurosurg Rev. 2025 Feb 25;48(1):265. doi: 10.1007/s10143-025-03344-0 (PMC11850463; doi:10.1007/s10143-025-03344-0)
Supplement: Supplementary file 1 — (DOCX 13.4 KB) [file 10143_2025_3344_MOESM1_ESM.docx]

**Collaborators - WorldWideWEB Investigators**

Ali Al Balushi MD^1^, Alex Brehm MD^2^, Rachel M. McLellan MS^3^, Kevin Premat MD^4^, Justin E. Vranic MD^3^, Jay A. Vachhani MD^5^, Vincent M. Tutino PhD^6^, Mohamed K. Ibrahim MD^7^, Marwa A. Mohammed MD^7^, Stéphanie Elens MD^8^, Julian Spears MD MSc^9^, Peter Sporns MD^2^, Erez Nossek MD^10^, Monika Killer-Oberfalzer^11^, Mohammad A. Aziz-Sultan MD^12^, Omer Doron MD PhD^3^, Sri Hari Sundararajan MD^1^, Yasuaki Inoue MD^3^, Shervin Mirshahi MD^3^, Cetin Imamoglu MD^12^, Ahmet Bayrak MD^12^

1 Department of Neurosurgery and Neuroradiology, New York Presbyterian Hospital and Weill Cornell School of Medicine, New York, NY, USA
2 Department of Neuroradiology, University Hospital of Basel, Basel, Switzerland
3 Neuroendovascular Program, Massachusetts General Hospital & Brigham and Women’s Hospital, Harvard University, Boston, MA
4 Department of Neuroradiology, Hôpital Pitié-Salpêtrière, Paris, France
5 Departments of Radiology & Neurosurgery, Cooper University Health Care, Camden, NJ, USA
6 Department of Neurosurgery, State University of New York at Buffalo, Buffalo, NY, USA
7 Departments of Radiology and Neurosurgery, Mayo Clinic, Rochester, MN, USA
8 Department of Neuroradiology , Hôpital Universitaire Erasme, Bruxelles, Belgium
9 Department of Neuroradiology, St. Michael’s Hospital, Toronto, ON, Canada
10 Departments of Radiology & Neurosurgery, NYU Langone Health Center, New York, NY, USA
11 Department of Neurosurgery, Christian Doppler University Hospital & Institute of Neurointervention, Salzburg, Austria
12 Department of Radiology, Medical Faculty of Ankara University, Ankara, Turkey
