# Supplementary material for: Incidence and predictors of Woven EndoBridge (WEB) shape modification following treatment of intracranial aneurysms in a large multicenter study
Source: Neurosurg Rev. 2025 Feb 25;48(1):265. doi: 10.1007/s10143-025-03344-0 (PMC11850463; doi:10.1007/s10143-025-03344-0)
Supplement: Supplementary file 2 — (DOCX 20.6 KB) [file 10143_2025_3344_MOESM2_ESM.docx]

**Supplementary table 1:** Cox Proportional Hazards regression model for no shape change versus minor shape change patients

| **Variable** | **Same**,  N = 237 (65%)*^1^* | **<50%**,  N = 127 (35%)*^1^* | Univariable Cox Regression | | Multivariable Cox Regression | |
| --- | --- | --- | --- | --- | --- | --- |
|  |  |  | **HR** **(95% CI)***^2^* | **p-value** | **HR** **(95% CI)***^2^* | **p-value** |
| Age (years) | 62 (56, 69) | 60 (50, 68) | 0.98 (0.97 to 1.00) | 0.052 | 0.99 (0.97 to 1.00) | 0.14 |
| Gender |  |  |  |  |  |  |
| F | 171 (72) | 94 (74) | — |  |  |  |
| M | 66 (28) | 33 (26) | 1.29 (0.86 to 1.94) | 0.22 |  |  |
| Smoking Status | 65 (28) | 39 (31) | 1.87 (1.26 to 2.77) | 0.002 | 1.83 (1.17 to 2.86) | 0.008 |
| Pretmt.mRSgroups |  |  |  |  |  |  |
| 3-5 | 17 (7.7) | 2 (1.6) | — |  |  |  |
| 0-2 | 204 (92) | 124 (98) | 5.62 (0.78 to 40.3) | 0.086 |  |  |
| Secondary Aneurysm Location |  |  |  |  |  |  |
| Anterior cerebral artery | 69 (29) | 48 (38) | — |  | — |  |
| Internal carotid artery | 37 (16) | 15 (12) | 1.21 (0.67 to 2.20) | 0.52 | 1.25 (0.60 to 2.61) | 0.55 |
| Vertebrobasilar artery | 51 (22) | 18 (14) | 1.00 (0.57 to 1.75) | >0.99 | 1.46 (0.79 to 2.73) | 0.23 |
| Middle cerebral artery | 80 (34) | 46 (36) | 0.79 (0.52 to 1.19) | 0.26 | 0.87 (0.53 to 1.45) | 0.6 |
| Ruptured aneurysm | 56 (25) | 28 (25) | 1.50 (0.97 to 2.30) | 0.067 | 1.18 (0.72 to 1.94) | 0.51 |
| Presentation Type |  |  |  |  |  |  |
| CN Palsy | 4 (1.8) | 2 (1.8) | — |  |  |  |
| Headache/Dizziness | 37 (16) | 12 (11) | 3.75 (0.48 to 29.3) | 0.21 |  |  |
| Incidental/Asymptomatic | 122 (54) | 70 (61) | 6.59 (0.89 to 48.6) | 0.064 |  |  |
| Recurrence | 1 (0.4) | 0 (0) | 0.00 (0.00 to Inf) | >0.99 |  |  |
| Ruptured aneurysm | 56 (25) | 28 (25) | 8.39 (1.12 to 62.7) | 0.038 |  |  |
| Seizures | 1 (0.4) | 0 (0) | 0.00 (0.00 to Inf) | >0.99 |  |  |
| Weakness/Numbness | 4 (1.8) | 2 (1.8) | 8.30 (0.73 to 93.9) | 0.087 |  |  |
| Prior Treatment | 13 (6.0) | 5 (4.4) | 0.91 (0.37 to 2.24) | 0.84 |  |  |
| Multiple Aneurysms | 76 (32) | 42 (33) | 0.92 (0.63 to 1.34) | 0.65 |  |  |
| Bifurcation Aneurysm | 198 (84) | 111 (87) | 0.90 (0.53 to 1.54) | 0.71 | 0.94 (0.47 to 1.90) | 0.87 |
| Branch Arising from Aneurysm | 26 (11) | 23 (18) | 0.73 (0.44 to 1.23) | 0.24 | 0.72 (0.38 to 1.40) | 0.34 |
| Secondary Aneurysm | 64 (30) | 30 (24) | 1.15 (0.76 to 1.75) | 0.51 | 1.33 (0.83 to 2.12) | 0.23 |
| Aneurysm Neck Size (mm) | 4.00 (3.30, 5.45) | 4.10 (3.25, 5.68) | 0.97 (0.86 to 1.10) | 0.68 |  |  |
| Maximum Aneurysm Diameter (mm) | 7.00 (6.00, 8.00) | 7.00 (6.00, 9.00) | 1.04 (0.97 to 1.12) | 0.31 |  |  |
| Aneurysm Width (mm) | 5.56 (4.30, 7.00) | 6.00 (4.65, 7.45) | 1.00 (0.93 to 1.08) | 0.92 |  |  |
| Aneurysm Height (mm) | 6.00 (4.90, 8.00) | 6.00 (5.00, 8.00) | 1.01 (0.94 to 1.09) | 0.78 |  |  |
| Height/width > 1.2 | 101 (43) | 45 (35) | 0.90 (0.62 to 1.30) | 0.59 | 0.79 (0.50 to 1.27) | 0.33 |
| Aspect > 1.5 | 100 (47) | 56 (44) | 1.06 (0.74 to 1.51) | 0.76 | 1.52 (0.97 to 2.36) | 0.065 |
| Dome/Neck | 1.35 (1.09, 1.64) | 1.33 (1.07, 1.65) | 1.14 (0.76 to 1.69) | 0.52 |  |  |
| Access Route |  |  |  |  |  |  |
| Femoral | 176 (74) | 123 (97) | — |  |  |  |
| Radial | 61 (26) | 4 (3.1) | 0.42 (0.15 to 1.15) | 0.091 |  |  |
| Immediate Flow Stagnation | 226 (95) | 104 (82) | 0.67 (0.42 to 1.07) | 0.1 | 0.66 (0.37 to 1.17) | 0.16 |
| WEB width - Aneurysm width ≤ 0.5 | 86 (36) | 51 (40) | 1.07 (0.74 to 1.54) | 0.72 | 1.12 (0.72 to 1.73) | 0.62 |
| Immediate occlusion: Remanent aneurysm | 124 (52) | 76 (60) | 1.26 (0.88 to 1.82) | 0.21 | 1.10 (0.70 to 1.71) | 0.68 |
| *^1^* Median (IQR); n (%) | | | | | | |
| *^2^* HR = Hazard Ratio, CI = Confidence Interval | | | | | | |

**Supplementary table 2: Treatment outcomes for no shape change versus minor shape change patients**

| **Variable** | **Same**,  N = 237 (65%)*^1^* | **<50%**,  N = 127 (35%)*^1^* | **P***^2^* |
| --- | --- | --- | --- |
|  |  |  |  |
| Thromboembolic Complications | 15 (6.3) | 8 (6.3) | >0.99 |
| Timing of Thromboembolic Complications |  |  | 0.19 |
| IO | 8 (57) | 7 (88) |  |
| PO | 6 (43) | 1 (13) |  |
| Duration of Thromboembolic Complications |  |  | 0.62 |
| Permanent | 4 (27) | 1 (13) |  |
| Transient | 11 (73) | 7 (88) |  |
| Hemorrhagic Complications | 5 (2.1) | 3 (2.4) | >0.99 |
| Timing of Hemorrhagic Complications |  |  | >0.99 |
| IO | 2 (40) | 2 (67) |  |
| PO | 3 (60) | 1 (33) |  |
| Duration of Hemorrhagic Complications |  |  | >0.99 |
| Permanent | 1 (20) | 1 (33) |  |
| Transient | 4 (80) | 2 (67) |  |
| Other Complications | 11 (6.4) | 4 (4.4) | 0.5 |
| Type of Other Complications |  |  | 0.31 |
| Air Embolization | 1 (10) | 0 (0) |  |
| Deployment issues | 5 (50) | 3 (75) |  |
| Puncture site hematoma/pseudoaneurysm | 4 (40) | 0 (0) |  |
| Vascular Dissection | 0 (0) | 1 (25) |  |
| Last Clinical Follow-Up | 11 (6, 17) | 15 (7, 27) | 0.002 |
| Last Modified Rankin Scale |  |  | 0.059 |
| 0 | 166 (75) | 104 (85) |  |
| 1 | 26 (12) | 10 (8.2) |  |
| 2 | 13 (5.9) | 1 (0.8) |  |
| 3 | 7 (3.2) | 6 (4.9) |  |
| 4 | 5 (2.3) | 0 (0) |  |
| 5 | 1 (0.5) | 0 (0) |  |
| 6 | 3 (1.4) | 1 (0.8) |  |
| Last Imaging Follow-Up | 9 (5, 15) | 13 (6, 23) | <0.001 |
| Immediate Raymond-Roy Classification |  |  | 0.1 |
| 1 | 57 (24) | 33 (26) |  |
| 2 | 56 (24) | 18 (14) |  |
| 3 | 124 (52) | 76 (60) |  |
| Last Follow-Up Occlusion |  |  | <0.001 |
| 1 | 167 (70) | 52 (46) |  |
| 2 | 47 (20) | 35 (31) |  |
| 3 | 23 (9.7) | 27 (24) |  |
| Last Follow-Up Raymond-Roy Classification |  |  | <0.001 |
| 1 | 168 (71) | 43 (38) |  |
| 2 | 46 (19) | 47 (41) |  |
| 3 | 23 (9.7) | 24 (21) |  |
| Retreatment Required | 12 (5.1) | 17 (14) | 0.004 |
| Type of Retreatment |  |  | 0.06 |
| Clipping | 3 (25) | 0 (0) |  |
| Endovascular techniques | 9 (75) | 17 (100) |  |
| *^1^* Median (IQR); n (%) | | | |
| *^2^* Wilcoxon rank sum test; Pearson’s Chi-squared test; Fisher’s exact test | | | |

**Supplementary table 3: Adjusted multivariable logistic regression for minor shape modification versus no shape modification (reference).**

| **Outcome** | **OR** **(95% CI)***^1^* | **p-value** |
| --- | --- | --- |
| **Retreatment** | 4.04 (1.29 to 14.8) | 0.022 |
| **Thromboembolic Complications** | 0.79 (0.28 to 2.19) | 0.66 |
| **Intracranial hemorrhage** | 3.50 (0.40 to 44.4) | 0.28 |
| **Inadequate Occlusion at last follow up** | 3.95 (1.69 to 9.91) | 0.002 |
| *^1^* OR = Odds Ratio, CI = Confidence Interval | | |
